# Emotional intelligence, psychological ownership, and digital resilience as mechanisms linking internal social media use to perceived organizational performance

**DOI:** 10.3389/fpsyg.2026.1883476

**Published:** 2026-08-03

**Authors:** Xiaoxia Li, Zehan Li, Baoguo Li

**Affiliations:** 1 Shandong Technology and Business University, Yantai City, Shandong Province, China; 2 Longkou Zhongyu Thermal Management System Technology Co., LTD, Longkou City, Shandong Province, China; 3 Department of Business Administration, Jeonbuk National University, Jeonju-si, Republic of Korea; 4 School of Business, Pingxiang University, Pingxiang, China

**Keywords:** creative engagement, digital resilience, emotional intelligence, perceived organizational performance, psychological ownership, social media use

## Abstract

This study examines how internal social media use (SMU) relates to employees' perceived organizational performance (ORP) by exploring the mediating roles of emotional intelligence, psychological ownership, creative engagement, and digital resilience. Grounded in the Resource-Based View and Social Exchange Theory, the study conceptualizes internal social media as a strategic digital resource that shapes employee psychological capabilities within contemporary workplaces. Data were collected from 792 full-time employees working in small and medium-sized enterprises in China using a cross-sectional, online survey. Structural Equation Modeling with bootstrapping was used to examine relationships among the constructs. The findings show that SMU is positively associated with emotional intelligence, psychological ownership, creative engagement, digital resilience, and perceived organizational performance. Moreover, all psychological constructs have significant indirect associations between SMU and ORP. The study contributes to digital workplace research by integrating psychological resource pathways into the examination of internal social technologies. It also offers practical insights, suggesting that organizations seeking to enhance performance should focus not only on technological investments but also on initiatives that cultivate emotional, psychological, and adaptive employee capacities.

## 1 Introduction

Digital transformation has changed the internal communications and development of employee competencies in organizations (Mohiya, 2025; Jiang and Luo, 2024; Madsen, 2023). One of the most significant tools of this change is internal social media use, such as DingTalk, Feishu, Slack, and Yammer. The platforms also allow employees to communicate more rapidly and effectively through knowledge sharing, real-time collaboration, and cross-level communication (Meira and Hancer, 2021; Men et al., 2023). In the context of Chinese SMEs where lack of resources, hierarchical organization, and fast digitalization make organizational dynamics unique, the internal social media use (SMU) has gained particular relevance (Wang et al., 2024). Although this is becoming more integrated, there is still a conceptual gap in how SMU is related to organizational outcomes (Men et al., 2020), and little empirical investigation regarding role of psychological factors has been conducted on it.

The literature provides conflicting perspectives on the role of internal social media in the performance of any organization. According to some scholars, SMU increases the efficiency of the communication process, the engagement of its employees, and promotes the collective intelligence that can be related to the organizational performance (Rasheed et al., 2021; Scott and Bruce, 1994; Wu, 2020). Others warn that social media can cause information overload, or even digital fatigue and this connection is not necessarily simple positive (Barney, 1991; Teece et al., 1997; Zhou and Yin, 2025). These contrasting findings indicate that the mechanisms through which internal social media relates to organizational outcomes remain insufficiently understood. More specifically, previous studies have generally adopted either technology-oriented perspectives that emphasize knowledge sharing and communication efficiency or social perspectives that focus on collaboration and employee interaction (Zhou et al., 2024; Kager et al., 2024; Mayer et al., 2004). While these studies have generated important insights, they frequently rely on single theoretical lenses or combine multiple theories without clearly explaining how the proposed mechanisms complement one another. Consequently, the processes through which internal social media is associated with employees' psychological capabilities and, ultimately, perceived organizational performance remain conceptually fragmented.

Methodologically, many existing studies have employed cross-sectional self-report survey designs and have primarily examined direct relationships between enterprise social media and outcomes such as knowledge sharing, employee engagement, or job performance (Madsen, 2023; Barney, 1991; Goleman, 1995). Comparatively fewer studies have simultaneously examined multiple theoretically distinct psychological mechanisms within a single integrated framework, particularly in the context of Chinese SMEs. This limitation restricts understanding of the different psychological pathways through which internal social media may be associated with perceived organizational performance.

The current research overcomes this drawback by using a two-theory framework in which the Resource-Based View (RBV) theory and the Social Exchange Theory (SET) are integrated. RBV introduces internal social media as a virtual asset, which is a digital resource that may not be valued until it is converted into employee capabilities that can hardly be replicated. SET is a complement to this, as it describes how online interactions, feedback, sharing of information, etc., create socio-emotional processes that can affect behavior in the workplace (Srivastava, 2013; Ertiö et al., 2024). The combination of these theories offers a focused and consistent rationale by assigning RBV the macro-level role of explaining why Internal Social Media Use constitutes a strategic organizational resource, while SET explains the micro-level relational processes through which this resource is associated with employees' psychological capabilities and perceived organizational performance.

On this basis, we conceptualize four critical psychological capabilities in a single framework. Emotional Intelligence that determines the way employees perceive and regulate emotions when dealing with digital communication (Mayer et al., 2004; Pierce et al., 2001). Psychological ownership, which is based on the feeling of responsibility and belonging that appears due to participative and open digital communication (Hamrick et al., 2024; Sugianto et al., 2023). Creative Engagement, which includes the willingness of the employees to ideate, innovate, and solve problems using internal social systems (Meira and Hancer, 2021; Blau, 1964). Digital resilience, the ability to respond to digital challenges, disruptions and changing technological demands (Nguyen et al., 2022; Weiss and Cropanzano, 1996). Such skills are especially essential in Chinese SMEs, where workers navigate a dynamic digital world, and it is common that they have little or no formal training, are subjected to hierarchical management and have strong cultural norms of harmony, obligation and group accountability. The social media within an organization might serve as a psychological enabler, which influences the manner in which employees work together, handle issues, manage emotional control, and react to digital stresses (Wang et al., 2024; Rasheed et al., 2021; Ashaye, 2023).

These psychological mechanisms are under-researched in context of SMU despite their significance. The available literature normally isolates them or puts a lot of emphasis on sharing of knowledge without investigating the role of various psychological processes in mediating the SMU-performance relationship together. Moreover, the psychological factors peculiar to the Chinese SMEs are rarely addressed in the prior research which influence the SMU adoption and perceptions among the employees. The present research fills these gaps by discussing the relation between internal social media use and these four psychological capabilities and how these capabilities are correlated with perceived organizational performance as well. The study utilized information gained from 792 employees working in various Chinese SMEs to answer the following research questions:

How internal social media use is related to perceived organizational performance in Chinese SMEs?

What is the connection between internal social media use and the emotional intelligence of the employees coupled with their creative participation, digital resilience, and psychological ownership?

How do these psychological capabilities relate to the perceived organizational performance?

Do these psychological abilities collectively represent the psychological channels in which internal social media usage is associated to perceived organizational performance?

This study makes contributions to the field of organizational psychology and digital management in multiple ways. First, it develops a more explicit integration of RBV and SET by distinguishing their complementary macro- and micro-level explanatory roles. Second, it simultaneously examines four theoretically distinct psychological mechanisms within a single parallel mediation framework rather than investigating each mechanism independently. Third, it extends enterprise social media research to the context of Chinese SMEs, where organizational culture and digital transformation create a distinctive setting for understanding employees' perceptions of organizational performance. Practically, the results inform the managers how to come up with social media systems that foster ownership, resilience, creativity and emotional intelligence.

## 2 Theoretical foundations

This paper relies on two complementary theoretical approaches namely the Resource-Based View (RBV) and the Social Exchange Theory (SET) in describing the relation between internal social media use (SMU) and employee psychological capabilities and its implication on the perceived organizational performance. These two theories have been chosen exclusively with the intention of addressing the issue of conceptual dilution in previous studies. This study does not consider RBV and SET as alternative and competing explanations, instead each theory is given a unique and complementary role to explain the association between Internal Social Media Use and perceived organizational performance.

In spite of this, previous research has either been based on RBV or SET. These perspectives are applied independently in general, resulting in an incomplete understanding regarding the impact of digital communication technologies on organizational performance. The RBV theory focuses mainly on the strategic importance of an organization's resources without giving much attention to the interpersonal and psychological dynamics by which the employees turn these resources into capabilities to improve organizational performance. By contrast, SET explains the reciprocity in social interaction and development of social relations but relatively little attention is given to why digital technologies are strategic resources for organizations. This theoretical distinction has left an important gap in understanding the mechanisms that links the internal social media use with Perceived Organizational Performance.

The present study adopts RBV as the macro-level theoretical foundation explaining why Internal Social Media Use constitutes a strategic organizational resource. However, RBV alone cannot explain how the value embedded within this technological resource is converted into employees‘ psychological mechanisms (Peck, 2018). This limitation motivates the integration of RBV with SET. When the Chinese SMEs are considered with limited financial and technological resources, it becomes crucial to develop the psychological mechanisms of the employees. According to RBV approach, internal social media platforms could be viewed as strategic intangible resources (Ribeiro et al., 2025). Such systems increase the visibility of communication, decrease the information asymmetry, and fasten the knowledge flows and facilitate the joint problem solving. RBV however highlights that technology cannot create organizational performance alone. Instead, it provides the strategic resource whose value depends on employees' ability to utilize it effectively (Yuan et al., 2024; Wu et al., 2025; Verma et al., 2023). Accordingly, RBV explains the source of potential competitive advantage but does not specify the interpersonal processes through which this potential is translated into employee psychological mechanisms. Therefore, in the present study, RBV serves exclusively as the macro-level explanation for why Internal Social Media Use represents a valuable organizational resource. The usefulness of digital tools varies with the success with which they are incorporated into the routine of employees, their competences and cognitive-emotional processes (Yuan et al., 2024; Wu et al., 2025; Verma et al., 2023). The four psychological abilities that are being conceptualized in this research, include emotional intelligence, creative engagement, and digital resilience and psychological ownership that aids in transforming the communicative potentials of Internal Social Media Use to relevant workplace actions. All these capabilities compose an integrated psychological capability framework which represents the impact of digital interactions on the emotional control of employees, feeling of responsibility, flexibility, and innovation orientation. RBV therefore justifies the notion that Internal Social Media Use can play a role in performance outcomes and how the psychological capabilities serve as processes under which this value is achieved. It explains the origin of organizational value (i.e., Internal Social Media Use as a strategic digital resource), but it does not fully specify the social and psychological mechanisms through which employees transform this resource into valuable organizational outcomes.

SET complements RBV by explaining the micro-level relational processes through which employees convert the strategic value of Internal Social Media Use into meaningful psychological resources. Whereas RBV explains why Internal Social Media Use possesses strategic potential, SET explains how reciprocal interactions, trust, feedback, recognition, and knowledge exchange occurring through these platforms cultivate employees' psychological capabilities. Thus, SET provides the missing interpersonal mechanism through which the resource-based advantages proposed by RBV become psychologically meaningful for employees (Wong and Law, 2002; Ashkanasy, 2024; Côté and Miners, 2006; Wang et al., 2024; Luqman et al., 2023). Within the SET framework, supportive digital interactions develop:

- Psychological ownership, by allowing participation, voice, and visibility in the processes of an organization.
- Emotional intelligence, by involving the employees to perceive the emotional clues in the digitally mediated communication and handle the interaction positively.
- Creative engagement, through fostering ideation and collaborative solutions in common online forums.
- Digital resilience, by subjecting employees to adaptive problem-solving and peer support in the face of digital issues.

Therefore, SET explains how the characteristics of digital communication relationships determine the psychological routes that could influence the perceptions of organizational performance by employees.

The integration of RBV and SET is the main theoretical contribution of this study. Rather than functioning as two parallel explanatory perspectives, the theories explain successive stages of the same process. RBV suggests that internal social media use is a strategic organizational resource processing latent value, while SET provides the understanding how regular social interaction through digital platforms allows employees to build their emotional intelligence, psychological ownership, creative involvement, and digital resilience. These psychological capabilities act as the processes that link the strategic value of internal social media use with perceived organizational performance. So, RBV helps to understand the importance of Internal Social Media Use whereas SET helps to understand how the value of Internal Social Media Use is realized as a result of employees' psychological development. Both theories are used together as a two-stage explanatory model to provide a clearer and more parsimonious interpretation of the proposed research model. This integrated view addresses an important theoretical gap that neither of the two frameworks can adequately explain independently.

## 3 Hypotheses development

The Internal Social Media Use (SMU) has completely transformed the way how employees communicate, cooperate, and interact in the organizations. Internal social media platforms in SMEs have become an important infrastructure to enable real-time interaction, cross level communication and prompt decision making (Luqman et al., 2023). Recent research also indicates that enterprise social media supports knowledge integration, employee collaboration and digital coordination in increasingly hybrid work environments (Chen et al., 2023; Ghorbanzadeh et al., 2021; Amabile, 1983). Based on Social Exchange Theory and Resource-Based View, these views contribute to articulate the interpersonal dynamics triggered by SMU as well as strategic value of ensuring the capabilities of employees. Internal Social Media Use enhances the transparency of information, limits the extent of delays in communications and ensures that employees have access to digital repositories, collaborative spaces and avenues of sharing knowledge (Kager et al., 2024). These platforms enable facilitative exchanges like feedback, recognition, and informational support. These exchanges reinforce the relationship ties, foster the expectation of co-operation and increase the number of employees in accordance with the organizational objectives. In the meantime, RBV theorizes Internal Social Media Use as an intangible digital asset that promotes coordination, responsiveness, and operational efficiency especially in SMEs with a scanty formalized structure (Ashaye, 2023). The previous studies indicate that SMU makes employees more aware of organizational actions and improves team coordination that can be used to develop better performance perceptions. Based on these theoretical and empirical findings, following hypothesis is developed:

*H1: Internal social media use is positively associated with perceived organizational performance*.

Digital communication conditions demand workers to perceive communication messages, reactions, and interactions that contain some emotional implications and respond to them accordingly (Goleman, 1995). The SMU platforms expose employees to a variety of affective interactions, including collaborative discussions, peer feedback, and digitally mediated social support, through which employees could be seeking assistance, offering of solace and providing feedback or overcoming miscommunication (Ribeiro et al., 2025). According to SET, emotional sensitivity and responsiveness can be strengthened by consistent and mutual contacts over the Internet (Amabile, 1983). The Chinese culture of the SMEs employees is characterized by emphasis on the relational harmony, saving face, and sensitive social behavior. These relational expectations are usually heightened by digital communication. Thus, employees who engage more frequently in digitally mediated interactions can develop emotional awareness and have better regulatory abilities.

*H2: There is a positive relation between internal use of social media and emotional intelligence of the employees*.

Psychological ownership develops when the workers develop a belief that they possess and individual ownership of the organizational objectives (Pierce et al., 2001). According to SET, the employees who see a possibility to exercise their voice, collaborate in the tasks, and be recognized in return to reciprocate the actions by becoming more attached to the organization. Social media within companies produce such participatory spaces by enabling transparent communication, collaborative knowledge sharing, and employee participation in organizational discussions, as increasingly documented in recent enterprise social media research (Wu et al., 2025). These affordances meet major psychological ownership antecedents of perceived control, meaningful involvement, and a sense of belonging. These effects can be enhanced in SMEs where employees tend to have direct communication with the decision-makers via digital media.

*H3: Internal social media use is positively associated with employees' psychological ownership*.

Creativity engagement demonstrates the readiness of the employees to devote energy, imagination, and cognitive capabilities to the development of new ideas. RBV implies that the Internal Social Media Use increase access to a variety of information and problem-solving knowledge whereas SET focuses on how supportive digital interactions encourage employees to contribute creatively in return (Zhou and Yin, 2025). Internal Social Media Use offer improved communicative visibility, so that employees can observe the way other people think and behave. It also improves the opportunities for quick ideation, discussion groups, and crowdsourced solutions. In Chinese SMEs, where innovation is becoming more digitally responsive, active users of the Internal Social Media Use can become more creative as they fulfill their tasks in a collaborative manner (Ashkanasy, 2024).

*H4: There is a positive relationship between internal social media use and the creative engagement of the employees*.

Digital resilience, the capacity to adjust to technological shocks, acquire new technology and to overcome digital adversities in the swiftly evolving digital settings (Nguyen et al., 2022). SET assumes that nurturing interactions like peer support, joint problem solving, and troubleshooting develop the confidence and flexibility of employees. Internal Social Media Use helps the staff to swiftly seek the advice of fellow employees, exchange digital solutions, and get real-time options when they encounter technological problems (Kager et al., 2024). Digital resilience represents a valuable employee capability that enhances the organizational agile and its operational continuity. In SMEs where formal digital training might be constrained, SMU can provide a necessary alternative to formal learning settings by providing employees the opportunity to develop resilience in an organic manner by being exposed to one another.

*H5: Internal social media use is positively associated with employees' digital resilience*.

Emotional intelligence has evolved into a mandatory psychological skill in digitally intensive work environments, where quick communication, cross-functional collaboration, and interaction with emotions are the most common situations (Ashaye, 2023). The staff that is characterized by a higher emotional intelligence can better read human signs, manage their emotions and have positive working relationships. In RBV approach, emotional intelligence is regarded as the valuable, rare and hard to imitate human resource that improves group functioning and organizational flexibility. SET also elaborates that emotionally intelligent workers create two-way trust, empathy and supportive interactions, relational processes which enhance team cohesion and provide flow of work easily (Peck, 2018). Emotionally intelligent employees can be useful in sustaining good social climates to minimize conflict and enhance coordination in Chinese SMEs, where work environments are often characterized by relational harmony (guanxi), respect, and collective effort (Ghorbanzadeh et al., 2021). Such benefits of relation are often directly symbolized in the perception of employees on the efficiency of organization, the quality of services provided, and the overall performance. The empirical research always demonstrates that emotional intelligence correlates with a better teamwork, quality of communication, conflict resolution, and job satisfaction, all have an effect on employees' ratings of organizational operation.

*H6: Emotional intelligence is positively related with perceived organizational performance by the employees*.

Psychological ownership indicates the degree to which employees experience a feeling of ownership, responsibility and emotional investment to their organization (Yuan et al., 2024). When employees feel that the organizational successes and failures are theirs, then increased commitment, discretionary effort, and stewardship behaviors are likely to be exhibited by the employees. Psychological ownership in the RBV perspective is a strategic human resource that promotes taking initiative, persistence, and alignment with the organizational values (Wu et al., 2025). To facilitate such a course, SET advocates the idea that as the employees feel that they are mutually supported, trusted, and have an opportunity to participate in the workplace they will reciprocate the action by deeper thinking and affective investment. This feeling of ownership encourages the workers to safeguard the interests of the organization, resolve issues independently, as well as demonstrate performance-favoring actions (Verma et al., 2023). In the case of SMEs, particularly in the fast changing and competitive environment of China, employees who possess high levels of psychological ownership often proffer suggestions, protect organizational properties and go an extra mile beyond the job scope, which might increase perceived performance of the organization.

*H7: There is a positive relation between psychological ownership and perceived organizational performance by the employees*.

Creative engagement is the psychological involvement of employees in new idea, experimentation of different solutions and use of imagination in enhancing work processes. RBV highlighted that creative engagement is an important employee skill that enhances innovation, problem-solving, and adaptability, which are important performance determinants (Amabile, 1983). SET also adds to this reasoning by stating that supporting relationships and mutual contacts stimulate the employees to devote the cognitive and emotional resources to creative work practices. Employees who are creatively engaged in the Chinese SMEs where innovation and flexibility are vital to staying competitive, are more likely to generate ideas that would increase efficiency, or improve the quality of the product or service offered (Wong and Law, 2002). The creative engagement can also enable the employees to react positively to resource constraint by finding other ways of meeting the organizational objectives. Many studies have associated the creative engagement with an increase in innovation, service quality, and operational effectiveness, which are direct contributors to perceptions of employees on organizational performance.

*H8: Creative engagement is positively linked to perceived organizational performance of the employees*.

Digital resilience is the ability of employees to adjust to technological changes, acquire the use of digital technologies effectively, and be productive despite difficulties related to digital processes (Ashkanasy, 2024; Côté and Miners, 2006). Digital resilience is an aspect of human capability within the RBV framework that allows the organizational ability to be adaptable to changes in technology and environmental alterations and be able to maintain operational continuity. According to SET, digital resilience emerges in the context where employees are supported with information, encouraged, and assisted with peers, which are the types of social exchange (Chen et al., 2023). With the fast digitalization that Chinese SMEs are undergoing, they often expose their employees to new mechanisms, system software upgrades, computerized coordination assignments, and technological limitations. The more digitally resilient ones can better preserve the stability of workflow, solve issues together, and incorporate new tools into the work process (Ghorbanzadeh et al., 2021). The studies show that strong employees have less trouble during the period of technological changes, maintain high performance when they are under pressure and generate team learning-all of which shape positive attitudes in the performance of organizations.

*H9: Digital resilience is positively correlated with employees' perceived performance of the organization*.

The Internal Social Media Use exposes the employees to regular online communication in terms of feedback, support requests, sentiments of appreciation, and emotional responses (Amabile, 1983). These exchanges have been theorized, using SET, to develop the emotional sensitivity and regulation abilities of employees. According to RBV, after developing emotional intelligence, it is an inherent ability and can be utilized in better organizational performance (Amabile, 1996). Thus, the workers, who are more often involved in internal digital communication, can feel that the performance of the organization is higher due to SMU that leads to the formation of emotional competencies promoting collaboration, flexibility, and positive behavior.

*H10: Emotional intelligence is a mediating factor between internal social media use and perceived organizational performance*.

Internal social media use offers exposure, involvement, and contribution channels, which SET defines as the basis of mutual commitment. The affordances are hypothesized to create psychological ownership, which will enable the employees to feel that they belong and that the results of the organization are partially theirs (Verkuyten, 2024). RBV emphasizes the concept of psychological ownership as a feature that enhances alignment and proactivity that can shape the perception of performance. Therefore, high team performance can be seen among the employees who actively engage in internal social media since these channels are linked with responsibility, emotional investment, and personal importance.

*H11: The psychological ownership has a mediating role between internal social media use and perceived organizational performance*.

The Internal Social Media Use can create a team-building atmosphere where employees share ideas and can discuss issues as well as engage in group brainstorming. According to SET, social exchange motivates employees to give feedback by making more cognitive and creative investments (Ahsan, 2023). RBV emphasizes creative involvement as a skill that can lead to innovativeness, efficiency and better output. Hence, workers who are more dependent on internal social media might feel that they are performing better since social media is linked to creativity that develops problem-solving and innovation abilities in teams.

*H12: The creative engagement is an associative route between internal use of social media and perceived performance in the organization*.

Internal social media facilitate instantaneous sharing of knowledge, sharing of troubleshooting and offering emotional assistance when online connections become disrupted (Amin and Wang, 2024). According to SET, positive social relations enable the staff to develop confidence and flexibility. RBV places digital resilience as a strategic ability to promote the stability and innovativeness of the operations within dynamic environments (Ashkanasy, 2024). Therefore, employees who sporadically use the social media provided within an organization can feel that the organization is performing better since digital resilience, which can be cultivated by informational and emotional interaction, is known to be connected to more adaptability and long-term productivity.

H13: *Digital resilience represents an associational pathway linking internal social media use with perceived organizational performance*.

**Figure 1:**
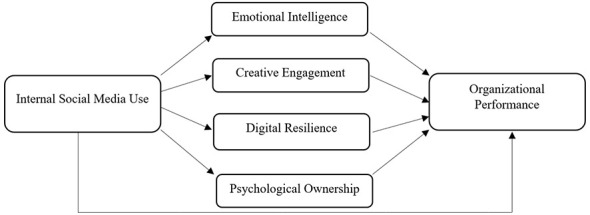
Conceptual framework.

The conceptual framework is shownin Figure 1.

The present study focuses on four mediating mechanisms—emotional intelligence, psychological ownership, creative engagement, and digital resilience—because they represent complementary yet conceptually distinct psychological resources through which internal social media use is associated with perceived organizational performance. Rather than reflecting overlapping constructs, each mediator captures a unique dimension of employees' psychological functioning. Although these constructs are positively related, they represent conceptually distinct psychological mechanisms through which Internal Social Media Use may be associated with perceived organizational performance. Thus, each construct represents a unique psychological domain—emotional regulation, motivational attachment, creative participation, and adaptive capability—that cannot be fully explained by the others. In the present study, these mediators are not regarded as alternative indicators for a broader positive emotional state, but rather as complementary mechanisms of various stages of employees' reactions to Internal Social Media Use. Because they are measured at the same time so they allow a complete understanding of how digital communication technologies are associated with organizational outcomes as compared with relying on a single higher-order construct like work engagement, perceived organizational support, or intrinsic motivation. Although these alternative constructs have importance in organizational behavior research, they mainly reflect general motivational or attitudinal states and do not represent the emotional, relational, creative and adaptive processes emphasized by the integration of RBV and SET.

## 4 Methods and materials

### 4.1 Study design and sample

The research design was quantitative and cross sectional to test the relation among internal social media use, psychological capabilities of employees and perceived organizational performance. The study involved 792 full-time workers of the SMEs in China. The participants were selected by non-probability purposive sampling method, which targeted the employees who use enterprise social media applications such as DingTalk, WorkWeChat, Microsoft Teams, or communication platform on the workplace. The Chinese SME context offers a unique cultural background to look into internal social media use. Collectivist values, relatively hierarchical organizational structures, and guanxi (interpersonal relationship networks) are some of the characteristics of many Chinese organizations that shape the ways in which communication, knowledge sharing, and mutual trust occur. These traditional norms are not being replaced but rather, enterprise social media platforms like DingTalk and WorkWeChat are typically integrated into the existing business environment, allowing users to continue with their workplace relationships while communicating in a more transparent, timely and cross-functional manner. Consequently, the Chinese SME context offers an appropriate setting for understanding how Internal Social Media Use relates to employees' psychological resources and perceived organizational performance.

The response rate was found to be 88 percent (792 valid responses out of 900 questionnaires sent out). Since the research was carried out among SMEs in China, the initial English-based questionnaire was translated to Mandarin through Brislin standardized technique of back-translation. The instruments were translated by two bilingual experts whose independent translation was then translated into Mandarin, with two other experts, who had no knowledge of the original scales, translating it once again into English. A pilot test was done on 35 workers working in the technology, retail and service industries to test the item clarity, cultural fit and internal consistency. Pilot comments suggested some slight changes in wording (e.g., changing Western centric phrases with Mandarin-related expressions appropriate at work), which were implemented prior to the full-scale administration. Prior to the survey, the participants were informed through a statement of informed consent of the purpose of the study, confidentiality of the research, execution of survey procedures and their right to withdraw throughout the whole process. No personal information or IP addresses and job record were gathered. The constructs were separated to form distinct sections, each was headed with a psychologically different title to minimize priming, and the descriptions were done in random sequence, and a wise neutral wording technique was employed to minimize evaluation apprehension. The survey took about 15 to 20 min to complete, and the survey platform did not allow a single participant to submit the survey more than once because the platform was able to recognize shared devices signatures.

### 4.2 Common method bias (CMB)

In order to mitigate the possibility of CMB, a number of procedural and statistical correctives were used. Procedurally, the respondents were assured complete anonymity to mitigate evaluation anxiety, the sections of the construction were divided with psychologically distinct headings, and the scale anchors were differed between the constructs in order to minimize the pattern of responses. The items were also randomized and the participants were well made aware of the fact that the management of the organization would not obtain individual responses. Harman single-factor test has shown that the first factor explained the 23.4 percent of the total variance, which was far below the 50 percent, indicating that no individual factor contributed to the total variance. Although these procedural and statistical remedies increase confidence that common method bias is unlikely to have substantially affected the findings, Harman's single-factor test alone is not sufficient to completely rule out common method variance. Therefore, variance inflation factors (VIFs) were also examined, and all VIF values were below the recommended threshold of 3.3, providing additional evidence that common method bias was not a serious concern. Nevertheless, because the data were collected from a single source at a single point in time, some degree of common method variance cannot be entirely excluded. Consequently, the findings should be interpreted with appropriate caution. Future research should employ longitudinal or multi-wave research designs, collect predictor and outcome variables from different sources, incorporate objective organizational performance indicators, and consider marker-variable techniques to further reduce the possibility of common method bias.

### 4.3 Instruments

The survey addressed six measures, namely, internal social media use, emotional intelligence, psychological ownership, creative engagement, digital resilience, and perceived organizational performance. In order to receive conceptual accuracy, cultural appropriateness, and psychometric rigor, the instrument was developed in multistage process that involved the process of scale selection, translation, pilot testing, and refinement of the validity. Measurements were done on a five-point Likert scale (1 = strongly disagree, 5 = strongly agree).

#### 4.3.1 Internal social media use (SMU)

The internal social media use was measured via 6-items scale adapted from Ou and Davison (Men et al., 2020). The scale measures the extent to which employees engage in platforms supported by the company in organizing their work, disseminating information, sharing knowledge, and communicating socio-emotional messages. The items emphasize:

- Collaboration and teamwork
- Information seeking and sharing
- Social connection and informal interaction
- Visibility and transparency of communication

#### 4.3.2 Emotional intelligence

Emotional intelligence was measured using the 12-items Wong and Law Emotional Intelligence Scale (WLEIS) (Wong and Law, 2002) through four sub-dimensions:

- **Self-emotion appraisal** – understanding one's own emotional states
- **Others' emotion appraisal** – ability to perceive and interpret others' emotions
- **Use of emotion** – leveraging emotions for performance, motivation, and decision-making
- **Regulation of emotion** – controlling disruptive emotions and managing stress

#### 4.3.3 Psychological ownership

Psychological ownership was assessed using the 7-items scale developed by Avey et al. (2009), capturing employees' subjective sense of possession and emotional investment in the organization. The scale evaluates:

- Sense of control (feeling empowered to influence work processes)
- Self-investment (contributing ideas, effort, and personal resources)
- Intimate knowledge and belongingness

In the context of Chinese SMEs—where collectivism, loyalty, and organizational identification are culturally salient—PO serves as a meaningful predictor of motivational attitudes and commitment.

#### 4.3.4 Creative engagement

Creative engagement was measured using 6-items adaptation from the study (Ahsan, 2023; Amin and Wang, 2024; Avey et al., 2009) combined with elements from Amabile's theory of creativity (Amabile, 1983, 1996). This construct reflects employees' psychological immersion in creative tasks, idea exploration, and problem-solving initiatives. Items capture both cognitive aspects (e.g., generating novel ideas) and affective aspects (e.g., enthusiasm for innovation).

#### 4.3.5 Digital resilience

Digital resilience was assessed using 6-items refined scale based on the study (Hobfoll, 1989). This measures employees' ability to:

- Adapt to new technologies
- Recover from digital disruptions
- Maintain performance during technological uncertainty
- Continue learning and adjusting strategies in evolving digital environments

#### 4.3.6 Perceived organizational performance

The performance of the organization was quantified through 5-items subjective performance measure that is highly used in literature (Avey et al., 2009; Hobfoll, 1989; Cropanzano et al., 2017; Hu and Bentler, 1999). Items evaluate the perception of employees regarding operational efficiency, innovation success, competitive position, and improvement. These measures are largely subjective, but prior research has shown that employees' performance perceptions are positively associated with objective organizational indicators, particularly when respondents are knowledgeable about their organization's operations and are directly involved in day-to-day work activities. Previous validation studies have also reported acceptable criterion-related validity, demonstrating moderate to strong positive correlations between subjective organizational performance measures and objective indicators such as sales growth, profitability, market share, and overall organizational effectiveness. Accordingly, subjective performance measures are widely accepted in organizational research when objective firm-level data are unavailable or difficult to obtain.

In the context of Chinese SMEs, obtaining standardized objective performance data is often difficult because of confidentiality concerns, variations in accounting and reporting practices, and the limited public availability of firm-level performance information. Consequently, employee perceptions provide a practical and theoretically justified proxy for Perceived Organizational Performance, as employees are well positioned to evaluate organizational functioning through their direct experiences with internal processes, collaboration, and operational outcomes. Moreover, the respondents in this study were full-time employees with substantial organizational tenure and regular engagement with enterprise social media platforms, increasing the likelihood that they possessed sufficient knowledge of organizational practices to provide informed assessments rather than relying solely on generalized organizational attitudes.

The scale was designed to emphasize relative performance, which further reduces the influence of industry- and firm-specific differences by asking respondents to assess their organization's performance relative to competitors rather than relying on absolute performance levels. Nevertheless, we acknowledge that employees' evaluations may still be influenced by favorable organizational attitudes or halo effects. Therefore, the findings should be interpreted as reflecting employees' perceptions of organizational performance rather than objective organizational outcomes. Future research should strengthen criterion validity by combining perceptual measures with objective or archival performance indicators and supervisor- or manager-rated performance measures wherever feasible.

### 4.4 Measurement model assessment

Before testing the structural model, a confirmatory factor analysis (CFA) was conducted to evaluate the adequacy of the six-factor measurement model. The model fit was assessed by using χ^2^/*df*, CFI, TLI, RMSEA and SRMR as per the recommended values proposed by Hu and Bentler (1999). Indicator reliability was confirmed by standardized factor loadings exceeding the recommended value of 0.70. Internal consistency was established using Cronbach's alpha and composite reliability values above 0.70. Convergent validity was supported by average variance extracted (AVE) values greater than 0.50, while discriminant validity was confirmed using both the Fornell–Larcker criterion and the heterotrait–monotrait (HTMT) ratio, with all HTMT values below the recommended threshold of 0.85. These results indicate that the measurement model possessed satisfactory reliability and construct validity prior to evaluating the structural relationships. Following confirmation of the adequacy of the measurement model, the structural model was estimated using Structural Equation Modeling (SEM) with maximum likelihood estimation. The bootstrapping procedure to estimate the indirect effects was performed with 5,000 resamples and bias-corrected confidence intervals to determine the mediation effects.

## 5 Empirical analysis

The descriptive statistics are reported in Table 1.

**Table 1:** Descriptive analysis (*N* = 792).

Variable	Category	Frequency	Percentage
Gender	Male	399	50.4%
	Female	393	49.6%
Age (in years)	18–25	117	14.8%
	Above 25–35	417	52.7%
	Above 35–45	193	24.4%
	More than 45 Years	65	8.2%
Industry sector	Technology	229	28.9%
	Education	133	16.8%
	Finance/Banking	144	18.2%
	Healthcare	107	13.5%
	Retail/Consumer Services	99	12.5%
	Logistics/Manufacturing	80	10.1%
Organizational tenure	Less than a year	97	12.2%
	1–3 years	279	35.2%
	Above 3 years−6 years	246	31.1%
	More than 6 years	170	21.5%
Social media usage	Less than a year	54	6.8%
	1–5 years	320	40.4%
	More than 5 years	418	52.8%

The distribution of this implies that the majority of the respondents were familiar with their organizational systems, culture, and practices of digital communication enough to complete their answers reliably and informed.

### 5.1 Reliability and validity

Before testing the hypothesized structural relationships, a Confirmatory Factor Analysis (CFA) was conducted to evaluate the adequacy of the six-factor measurement model. The measurement model demonstrated a satisfactory fit to the data (χ^2^/*df* = 2.21, CFI = 0.97, TLI = 0.96, GFI = 0.94, RMSEA = 0.041, SRMR = 0.034), indicating that the proposed measurement structure adequately represented the observed data according to the recommended thresholds proposed by Hu and Bentler (1999).

Table 2 gives the results of reliability and convergent validity of all six latent constructs measured in the study. Even in the measurement model, the items had high standardized factor loadings of between 0.76 and 0.87, which is more than the recommended minimum of 0.70 to provide sufficient indicator reliability. A further test on high internal consistency is Composite Reliability (CR) and Cronbachs Alpha (CA) which have values of between 0.907 and 0.955 and 0.86 and 0.91 respectively, which are far above the accepted threshold of 0.70. Convergent validity which is determined by Average Variance Extracted (AVE) also passed the recommended criteria in that, the AVE values ranged between 0.654 and 0.716 implying that all constructs accounted more than half the variance in their respective items of measurement (Avey et al., 2009). The combination of these results proves that this is a robust and valid model of measurement, which forms the basis of the psychometric model of further testing.

**Table 2:** Reliability analysis.

Variables	Item	Loading	CR	CA	AVE
Internal social media use (SMU)	SMU1	0.82	0.919	0.87	0.654
	SMU2	0.84			
	SMU3	0.79			
	SMU4	0.76			
	SMU5	0.81			
	SMU6	0.83			
Emotional intelligence (EIN)	EIN1	0.85	0.955	0.91	0.716
	EIN2	0.87			
	EIN3	0.81			
	EIN4	0.78			
	EIN5	0.83			
	EIN6	0.80			
	EIN7	0.86			
	EIN8	0.77			
	EIN9	0.84			
	EIN10	0.82			
	EIN11	0.79			
	EIN12	0.83			
Psychological ownership (PSO)	PSO1	0.84	0.931	0.90	0.675
	PSO2	0.86			
	PSO3	0.82			
	PSO4	0.80			
	PSO5	0.81			
	PSO6	0.79			
	PSO7	0.83			
Perceived organizational performance (ORP)	ORP1	0.81	0.907	0.86	0.660
	ORP2	0.84			
	ORP3	0.80			
	ORP4	0.82			
	ORP5	0.79			
Digital resilience (DGR)	DGR1	0.82	0.928	0.89	0.681
	DGR2	0.84			
	DGR3	0.79			
	DGR4	0.83			
	DGR5	0.85			
	DGR6	0.80			
Creative engagement (CRE)	CRE1	0.83	0.936	0.90	0.686
	CRE2	0.84			
	CRE3	0.81			
	CRE4	0.79			
	CRE5	0.85			
	CRE6	0.82			

CR, construct reliability; CA, Cronbach's alpha; AVE, average variance extracted.

Discriminant validity findings are summarized in Table 3.

**Table 3:** Discriminant validity.

Variable	Mean	SD	SMU	EIN	PSO	ORP	DGR	CRE
SMU	3.47	0.77	0.81					
EIN	3.82	0.66	0.48	0.85				
PSO	3.63	0.71	0.44	0.53	0.82			
ORP	3.71	0.74	0.41	0.46	0.49	0.81		
DGR	3.76	0.73	0.45	0.50	0.47	0.52	0.83	
CRE	3.76	0.70	0.47	0.52	0.50	0.54	0.55	0.83

The diagonal elements in Table 3 represent the square roots of the AVE for each construct and are greater than the corresponding inter-construct correlations, thereby satisfying the Fornell–Larcker criterion for discriminant validity. Furthermore, all Heterotrait–Monotrait (HTMT) ratios were below the recommended threshold of 0.85, providing additional evidence that each construct captures a distinct theoretical concept. These findings demonstrate satisfactory discriminant validity of the measurement model.

### 5.2 Structural equation model (SEM)

Following confirmation of the adequacy of the measurement model, the hypothesized structural model was estimated using Structural Equation Modeling (SEM) with maximum likelihood estimation. In addition to model fit, the explanatory power of the model was assessed using the coefficients of determination (*R*^2^) for the endogenous constructs. The model shows a satisfactory explanatory capability of the proposed theoretical model. The structural model was then tested for all hypothesized paths. The standardized path coefficients, *t*-values, and 95% confidence intervals are shown in Table 4, which used a bootstrapping procedure with 5,000 bootstrapping resamples.

**Table 4:** SEM results.

Hypotheses	Path coefficient	*t*-values	95% CI	p-value	Result
H1:SMU → ORP	0.28	5.21	[0.17, 0.37]	0.001	Supported
H2:SMU → EIN	0.56	10.24	[0.47, 0.64]	0.001	Supported
H3:SMU → PSO	0.51	5.89	[0.31, 0.60]	0.003	Supported
H4:SMU → CRE	0.47	5.66	[0.30, 0.55]	0.002	Supported
H5:SMU → DGR	0.44	5.72	[0.28, 0.46]	0.003	Supported
H6:EIN → ORP	0.37	7.02	[0.27, 0.45]	0.001	Supported
H7:PSO → ORP	0.39	7.46	[0.28, 0.46]	0.001	Supported
H8:CRE → ORP	0.33	5.12	[0.20, 0.41]	0.003	Supported
H9:DGR → ORP	0.30	5.41	[0.18, 0.38]	0.002	Supported
H10:SMU → EIN → ORP	0.207	4.78	[0.14, 0.27]	0.001	Partial mediation
H11:SMU → PSO → ORP	0.204	4.66	[0.13, 0.27]	0.001	Partial mediation
H12:SMU → CRE → ORP	0.162	4.18	[0.10, 0.22]	0.003	Partial mediation
H13:SMU → DGR → ORP	0.138	3.96	[0.07, 0.20]	0.002	Partial mediation

(χ^2^/*df*) = 2.21; CFI = 0.97; TLI = 0.96; GFI = 0.94; RMSEA = 0.041; SRMR = 0.034.

Results of the SEM show that all four psychological mechanisms such as emotional intelligence, psychological ownership, creative engagement and digital resilience were positively associated with the internal social media use. The psychological resources, in turn, were positively associated with perceived organizational performance. The findings indicated that emotional intelligence, psychological ownership, digital resilience, and creative engagement were positively associated with perceived organizational performance. These results suggest that perceived organizational performance is not only related to internal social media use, but also with psychological states related to the use of social media. Moreover, all four psychological constructs partially mediated the relationship between internal social media use and perceived organizational performance, highlighting that the link between social media use and performance is direct and indirect. In addition, mediation analysis indicates that the positive relationship between internal social media use and perceived organizational performance is stronger when social media use is associated with higher emotional intelligence, psychological ownership, creative engagement, and digital resilience. Finally, the model showed a good fit for all the indices, justifying the validity and predictive power of the proposed theoretical framework.

The structural model's explanatory power was also assessed by the coefficient of determination (*R*^2^). The results show that internal social media use accounts for 31% of the variance in the emotional intelligence (*R*^2^ = 0.31), 26% of the variance in psychological ownership (*R*^2^ = 0.26), 22% of the variance in creative engagement (*R*^2^ = 0.22) and 19% of the variance in digital resilience (*R*^2^ = 0.19). The proposed model accounted for a significant proportion of the variance in perceived organizational performance (*R*^2^ = 0.64) collectively.

## 6 Discussion

This paper examined the relationship between internal social media use and perceived organizational performance through the psychological mechanisms: emotional intelligence, psychological ownership, creative engagement and digital resilience. The findings need to be contextualized in the cultural context of Chinese SMEs. The Chinese workplace environment emphasizes hierarchical relationships, respect for authority, and guanxi, in which interpersonal trust and relationships are important factors in organizational communication. These cultural features cannot be diminished by Internal Social Media Use, but can be enhanced by extending the relationship building opportunities, improving knowledge across hierarchical boundaries, and supporting collaboration without altering the organizational rules and regulations. Thus, the positive links found in this study should be seen as a result of the interaction between enterprise social media affordances and the underlying relational culture of the Chinese SMEs, and not the technology itself. This cultural context could be a contributing factor to the positive relationship of Internal Social Media Use with psychological ownership, emotional intelligence, creative engagement, and digital resilience because digital interaction could reinforce formal communication within organizational structures and informal communication with one another. The results suggest that Internal Social Media Use is not just a communication tool, but an organizational communication resource that is associated with employees' behavior and perceived organizational outcomes (Hobfoll, 1989). These findings reveal that there is a positive association between the use of social media and perceived organizational performance. Those employees who reported more active use of internal social media platforms also reported greater coordination, sharing of knowledge, and information access (Cropanzano et al., 2017). The findings are comparable to those of previous researchers, who indicated that enterprise social media was associated with improved collaboration, cross-functional problem-solving, and efficiency in communication (Mohiya, 2025; Chen et al., 2023; Amabile, 1996). This research builds upon the existing body of literature by revealing that these performance associations are not only operational but are also partially explained by psychological mechanisms, suggesting that digital platforms are associated with the development of employee competencies and motivation (Hu and Bentler, 1999).

The analysis showed that Hypothesis 2 was supported since emotional intelligence is positively related to the use of social media. Based on Social Capital Theory, social interactions on internal platforms may help employees interpret social cues, emotional responses, and navigate digital interactions (Amabile, 1983; Verkuyten, 2024; Avey et al., 2009). Regular interaction may be associated with the development of empathy, self-awareness, and adaptive social behavior, which are basic emotional competencies linked to organizational performance (Caya and Mosconi, 2023; Ashaye et al., 2023). These findings suggest that Internal Social Media Use may facilitate emotionally supportive interactions that strengthen employees' capacity to regulate interpersonal communication in digitally mediated workplaces.

The third hypothesis was also confirmed, indicating that active participation in internal social media is positively associated with a stronger feeling of ownership. Participatory decision-making, content contribution, and recognition are among the mechanisms associated with stronger feelings of control and identification of employees with organizational goals (Barney, 1991; Bodhi et al., 2024). These findings are consistent with the notion that opportunities for participation and visibility within enterprise social media strengthen employees' psychological attachment to the organization. Rather than suggesting that these platform affordances automatically generate psychological ownership, the findings indicate that they provide opportunities through which employees may develop stronger feelings of ownership when supportive organizational conditions are present. This highlights the importance of social media in being associated with the development of such intrinsic commitment, and not merely information sharing.

Hypothesis 4 suggested that creative engagement is positively associated with the internal social media use. This is aligned with Componential Theory as it emphasizes that creativity thrives when conditions are conducive to intrinsic motivation, skill application and facilitative resources. Design and governance are important factors to consider, as social networks should not inhibit, but support creativity (Dahlawi et al., 2025). Such opportunities will not impact all employees in the same manner, since employees' intrinsic motivation and organizational innovation and psychological safety depend on creative engagement.

According to Hypothesis 5, there was a positive relationship between digital resilience and internal social media use. Access to informational and social resources within internal social media could assist in adjusting to technological disruptions. More digitally resilient employees may adapt the platform changes, cyber-attacks, or working remotely (Charbaji et al., 2025). Managers can support these associations by integrating digital tools with problem-solving communities and resilience training. These findings further suggest that enterprise social media may function as an informal learning environment through which employees continuously develop adaptive digital capabilities.

Emotional intelligence, psychological ownership, creative engagement, and digital resilience were all positively related to perceived organizational performance through the four psychological mechanisms. Employees with higher emotional intelligence may be better able to overcome stress, manage conflicts, and work together, which is associated with higher perceived organizational performance (Zhou and Yin, 2025; Dwivedi et al., 2022). Psychological ownership is positively associated with discretionary effort, initiative, and organizational citizenship behavior, which are linked to improved collective outcomes (Amin and Wang, 2024; Men et al., 2023). Creative engagement is positively associated with innovation in tasks, process improvement, and problem-solving (Cropanzano et al., 2017; Hair et al., 2019). Organizations may strengthen these positive associations through providing safeguarded periods of ideation, recognition opportunities, and knowledge-sharing platforms. Digital resilience is positively associated with continuity during disruptions and may enable employees to take advantage of emerging opportunities, although its association with performance may be more salient in unstable situations than in stable situations (Ertiö et al., 2024).

It was found that all four psychological mechanisms partially mediate the relationship between the use of social media and perceived organizational performance (H10–H13). These findings indicate that social media is not just a technological channel, but is associated with the development of psychological abilities linked to performance (Dwivedi et al., 2022). The most significant mediating pathways were emotional intelligence and psychological ownership, which suggest that the human–digital interface may play an important role in the relationship between platform interactions and perceived organizational performance (Luqman et al., 2023; Kim, 2024). Creative engagement and digital resilience are also important mediating pathways, suggesting that enterprise social media should be viewed as a platform that simultaneously supports multiple psychological capabilities, each contributing a distinct pathway linking digital interaction with perceived organizational performance. These mediating relationships should not be understood as universal or inevitable, but as context-dependent relationships that can differ in the organizational culture, communication climate, leadership practices and employee characteristics. For practice, organizations should consider building platform implementation along with other components of the supporting systems—learning opportunities, recognition, governance and culture—to achieve the maximum benefits associated with Internal Social Media Use.

### 6.1 Theoretical implications

The study has a number of significant theoretical contributions, by combining the perspectives of the Resource-Based View and the Social Exchange Theory to establish how the internal use of social media is associated with the performance of organizations via the psychological mechanisms. Regarding the RBV, this study proves that SMU is a valuable resource of the organization that is linked with the intangible skills of employees' emotional intelligence, psychological ownership, creative involvement, and digital resilience. The research demonstrates through empirical evidence, that these capabilities have a mediating role in the relation between SMU and perceived organizational performance. This is in line with the premise of RBV that sustained competitive advantage comes from the exploitation of internal capabilities and human capital that are valuable, rare, and hard to copy. Therefore, the paper expands the theory of RBV by defining digital communication platforms as resources-creating enablers with psychological and cognitive resources as key mediator variables in the value-creation process.

In the perspective of Social Exchange Theory, the findings demonstrate that the participation of employees in social media platforms contributes to performance and a healthy psychological condition. Through augmented SMU, the employees are stronger in their emotional intelligence and their psychological ownership and this makes them work harder, more creatively and dedicate themselves to the organization. This two-way exchange notion validates the main principle of SET that organizational resources are reciprocated by the employees in the form of improved discretionary behaviors, including creative involvement and resiliency. Connecting SMU to employee readiness to engage in activities beyond the official job expectations, the study builds upon the contexts of SET that it defines digital communication tools as a contextual process which inducts the social exchange relationships and creates a mutually beneficial outcome between the employees and the organization.

### 6.2 Practical implications

The study also offers practical guidance for managers regarding Internal Social Media Use to improve organizational performance. The managers can invest in training programs to develop digital empathy, feedback management and conflict resolution training. Ownership can be developed by means of system features, which enable employees to share ideas, involve them in the decision-making process and obtain recognition. Managers should establish voice mechanisms, recognition badges, and open sharing of information to improve the identification of employees.

Internal Social Media Use should be designed deliberately in a way that it can be ideated, cross-functional and knowledge recombination. Innovative behavior toward performance can be encouraged by providing secure time to be creative, submitting ideas and recognition systems. Managers can set up support networks for their employees, including peer networks, problem-solving groups, and stress management training, to prepare them for technological shifts and disruptions. The results indicate that the interventions are not supposed to be independent and should be multi-faceted (that is, encourage creativity, resilience, emotional skills and ownership). The deployment of the platform must be complemented by culture, governance, learning infrastructure and reward systems, to maximize the benefit of Internal Social Media Use on performance. When formulating their strategies toward SMEs in China, managers may consider the limitations of resources, different levels of digital literacy, and Chinese culture that affect adoption. Barriers can be countered with personalized implementation plans, gradual introduction, and constant feedback, which can make the platform even more effective. In addition to operational communication, Internal Social Media Use is potentially an important strategic organizational resource that promotes the development of human capital. Platform design, employee capability-building and supportive culture can be long-lasting and provide performance advantages, especially in dynamic or uncertain environments.

### 6.3 Limitations and future research

Although this research is valuable in terms of offering insights into the psychological processes that are associated with the relationship between internal use of social media and organizational performance, there are some limitations that should be taken seriously. The research was a cross-sectional survey based on self-reported data of employees. As a result, causal inferences cannot be made with a lot of conviction. The association between social media use and the psychological processes and organizational performance should be treated as a relationship and not as a strong causal impact. Future studies may utilize longitudinal, experimental or multi-wave research designs to study the dynamics over time and develop causal directionality.

Although the study adopted the statistical remedies in the form of Harman one-factor test and VIF analysis, the single source, same time survey design increases the chances of CMB. Collecting all variables from the same respondents at one point in time may have inflated the observed relationships because of common rater effects, consistency motifs, or social desirability bias. Future research should adopt research designs that reduce common method variance, such as collecting predictor, mediator, and outcome variables at different time points (multi-wave designs), obtaining organizational performance evaluations from supervisors or organizational records, combining self-reported and objective data sources, or incorporating marker variables and other procedural remedies.

Although the research showed a reasonable reliability and convergent and discriminant validity, some slight discrepancies between the past reports were also found. The future research should provide excellent psychometric validation of all constructs with the possibility of using qualitative pretesting or cognitive interview to enhance content validation. The research is targeted at the SMEs in China, which might not be generalized to other organizational or national setups. The adoption of social media and the psychological impacts may depend on cultural issues, the availability of resources, and the digital infrastructure.

Future research should also examine whether the observed relationships differ across cultural contexts, as the influence of Internal Social Media Use may vary according to national culture, organizational hierarchy, and relationship-oriented workplace norms such as *guanxi*. Comparative cross-cultural studies would help determine the generalizability of the present findings.

## 7 Conclusions

This research explored the relation of internal social media use with the organizational performance through mediating roles of emotional intelligence, psychological ownership, creative engagement, and digital resilience. Empirical evidence shows that there are both direct and indirect relations of Internal Social Media Use with perceived organizational performance. The internal use of social media positively associated with emotional intelligence, psychological ownership, creative engagement, and digital resilience, realizing that the use of digital platforms is positively associated with emotional, psychological, and adaptive resources of employees. These mechanisms of psychology are linked with the performance of the organization, which proves them as the key mediators in relationship of digital interaction and real company gains. The mediation results also indicate that even though the use of social media has a direct relation with performance, it has the indirect relation with psychological resources. The emotional intelligence and the psychological ownership are the strongest mediators and it may be assumed that human-digital interface is the key toward the actualization of organizational value. This relationship is also mediated by creative engagement and digital resilience, which illustrates the significance of multi-faceted and integrative processes between technology and performance.

Theoretically, this research contributes by merging various psychological concepts into the field of digital workplace literature and explains how Internal Social Media Use serves as a tool of creating intangible human capital resources. It builds on RBV and Social Exchange Theory indicating that emotional, cognitive, and behavioral resources developed through digital engagement constitute strategically valuable organizational resources. The multi-mediator model shows that benefits of the performance are functions of psychological processes, which provides a more detailed look at the development of human resources with the help of technology.

Practically, the results highlight the fact that managers should not rely to use technology deployment as the only tool. To make the optimal value of Internal Social Media Use, it is necessary to have strategic design and supportive interventions, including training to increase emotional intelligence and digital empathy, rewarding mechanisms and involvement aspects in building psychological ownership, platforms and processes that foster creative engagement and knowledge sharing, programs to create a resilience and flexibility that occurs especially when there is a technological or organizational crisis.

In conclusion, this paper showed that internal social media use is associated with psychological and behavioral resources that is linked with the performance of an organization. Through strategic deployment of digital tools, including emotional intelligence, ownership, creativity, and resilience, organizations, and especially SMEs, can develop more engaged workforces, more adaptive workforces, and high-performing workforces that are able to endure through current digital transformation.

## Data Availability

The original contributions presented in the study are included in the article/supplementary material, further inquiries can be directed to the corresponding author.
